# Differentially expressed genes in preimplantation human embryos: potential candidate genes for blastocyst formation and implantation

**DOI:** 10.1007/s10815-016-0745-x

**Published:** 2016-05-30

**Authors:** Erika M. Munch, Amy E. Sparks, Jesus Gonzalez Bosquet, Lane K. Christenson, Eric J. Devor, Bradley J. Van Voorhis

**Affiliations:** 1 Department of Obstetrics and Gynecology, The University of Iowa Carver College of Medicine, 200 Hawkins Drive, PFP 31330, Iowa City, IA 52242 USA; 2 Department of Molecular and Integrative Physiology, The University of Kansas School of Medicine, Kansas City, KS 66160 USA

**Keywords:** Preimplantation embryo, Gene expression, Blastocyst, Embryo culture, Cancer

## Abstract

**Purpose:**

The aim of this study was to determine which genes and gene pathways are differentially expressed when comparing human blastocysts with cleavage-stage embryos.

**Methods:**

We individually assessed gene expression in preimplantation human embryos at cleavage (*n* = 3) and blastocyst (*n* = 3) stages. Gene expression patterns were then validated in publically available datasets and then independently validated in vitro with additional human embryos using TaqMan gene expression assays. Immunolocalization studies were conducted to identify protein expression in intact blastocyst-stage embryos.

**Results:**

Compared to cleavage-stage embryos, blastocyst-stage embryos differentially expressed 51 genes (*p* < 0.001), with overrepresentation in amoebiasis pathways and pathways in cancer. Of these 51 genes, 21 were found to be independently validated in a separate, publically available dataset, with a substantial agreement with our initial findings (κ = 0.8). In an independent set of cleavage- and blastocyst-stage embryos, we validated that six of eight tested genes were differentially expressed (*p* < 0.05) by RT-qPCR. Immunofluorescence studies documented the presence of two studied proteins in the trophectoderm of blastocyst-stage embryos.

**Conclusions:**

Differentially expressed genes may be implicated in the invasion and proliferation of the early embryo. Our research highlights specific genes that may be further studied for their role in the implantation process and additionally raises questions about localized gene and/or protein expression in the trophectoderm, which could affect protocols for, and interpretation of, trophectoderm biopsies performed in in vitro fertilization cycles.

**Electronic supplementary material:**

The online version of this article (doi:10.1007/s10815-016-0745-x) contains supplementary material, which is available to authorized users.

## Introduction

Human embryonic genome activation begins between the two- and eight-cell stages [1, 2]. While stored maternally derived oocyte RNA transcripts are necessary for the nascent embryo to complete the first mitotic divisions, continued embryonic development is predicated on the expression of embryonic genes which lead to blastocyst formation and, ultimately, result in successful implantation.

Our understanding of genes critical to embryonic implantation remains limited. Early studies of gene expression in preimplantation embryos examined differential expression as early as prefertilization oocytes through as late as blastocyst-stage embryos and even embryonic stem cells [3, 4]. These studies have identified genes important in the transition from maternal to embryonic genome expression [2, 5–7], including those involved in DNA repair [8], cellular growth [9], trophectoderm development [10], as well as novel RNA sequences never seen in other human cell types [11]. However, such large profiling studies often make very broad comparisons with very different cell types (such as oocytes with blastocyst-stage embryos or cleavage-stage embryos with established human embryonic stem cell lines). Additionally, the abundance of raw data generated in such studies makes it difficult to meaningfully interpret all data in order to more deeply understand physiologic processes occurring in between these points in development. Specifically, these studies have not thoroughly examined the role of gene expression in blastocyst formation and function, with arguably the most important of these functions being successful implantation and pregnancy.

Here, we identify genes differentially expressed in human blastocyst-stage embryos as compared to cleavage-stage embryos and timepoints chosen for the purpose of identifying changes occurring after the onset of embryonic genome expression. We suggest that genes with differential expression in blastocyst-stage (as compared to cleavage-stage) embryos are likely to have significance both in the structure and the function of the blastocyst. The differentially expressed embryonic genes reported here, validated both in silico and in vitro, are associated with cellular movement and cancer pathways, both with shared characteristics of an implanting and invading embryo. Using immunofluorescence, we localized two of these proteins, S100A14 and S100A16, to the trophectoderm.

## Materials and methods

### Acquisition and culture of embryos

Approval from the University of Iowa’s Institutional Review Board (#200804752) was obtained prior to the performance of all experiments. Embryos used in our study had been specifically donated for use in research (#200109085) by patients who had undergone in vitro fertilization treatment, using either conventional insemination or intracytoplasmic sperm injection, and had supernumerary zygotes cryopreserved at the pronuclear stage. These embryos were cryopreserved and thawed as described previously [12]. For all experiments, embryos (*n* = 136) were cultured using commercially available embryo culture media supplemented with 20 % Quinns Advantage Serum Protein Substitute (Cooper Surgical) in 5.5–6.0 % CO_2_ in air at 37 °C. Embryos designated for study on the third day post fertilization (D3) were cultured for 48 h, whereas embryos designated for study on the fifth day post fertilization (D5) were cultured for 96 h. All D3 embryos included in the studies (*n* = 27) exhibited 6–10 blastomeres with ≤20 % fragmentation, while D5 blastocyst-stage embryos selected for study (*n* = 27) were blastocysts with at least an “A” or “B” rating for both the trophectoderm and inner cell mass scores, as graded using the classification system outlined by Gardner and Schoolcraft [13].

### Discovery via microarray: hybridization, microarray construction, and quality control

For the initial discovery set, RNA was isolated from each of six human embryos (three D3 and three D5, processed individually) using the RNeasy kit (Qiagen) according to the manufacturer’s protocol. Total RNA was converted to signaling pathway impact analysis (SPIA)-amplified complementary DNA (cDNA) using the WT-Ovation Pico RNA Amplification System, v1 (NuGEN Technologies) according to the manufacturer’s recommended protocol. The SPIA-amplified cDNA product was purified through a Qiagen QIAquick PCR Purification column (Qiagen) according to modifications from NuGEN. SPIA-amplified DNA (4 μg) was used to generate sulfotransferase (ST) cDNA using the WT-Ovation Exon Module v1 (NuGEN Technologies) and cleaned with the Qiagen column as above. This product (5 μg) was fragmented (average fragment size = 85 bases) and biotin-labeled using the NuGEN FL-Ovation cDNA Biotin Module, v2 (NuGEN Technologies) as per the manufacturer’s recommended protocol. The resulting biotin-labeled cDNA was mixed with Affymetrix eukaryotic hybridization buffer, placed onto Affymetrix Human Exon 1.0 ST arrays, and incubated at 45 °C for 18 h with 60 rpm rotation in an Affymetrix GeneChip Hybridization Oven, model 640. Following hybridization, the arrays were washed, stained with streptavidin-phycoerythrin (Molecular Probes, Inc.), and signal-amplified with antistreptavidin antibody (Vector Laboratories, Inc.) using the Affymetrix Fluidics Station, model 450. Arrays were scanned with the Affymetrix scanner (model 3000) with 7G upgrade, and data were collected using the GeneChip operating software (GCOS v3.1).

### Gene expression and pathway analysis

CEL file output from Affymetrix microarray processing was uploaded, normalized within each array using endogenous expression and array exposure controls, and filtered if excessive missing values or outliers were detected. Probe set summaries and annotations were abstracted from standard annotation files specific to Affymetrix Human Exon 1.0 ST arrays (http://www.affymetrix.com/support/technical/byproduct.affx?product=huexon-st). Genes were considered to be differentially expressed when differences achieved a univariate significance level of *p* <0.001, to avoid false-positive results due to multiple comparisons. To identify overrepresented and significant pathways among the selected significant genes in the Kyoto Encyclopedia of Genes and Genomes (KEGG) [14], we used MetaCore (GeneGo, Inc.) and clusterProfiler, an R statistical package. Both are integrated and curated *knowledge-based* platforms for pathway analysis. To include all possible overrepresented pathways, the enrichment analysis included all differentially expressed genes significant at a *p* value <0.01, with a false discovery rate (FDR) of <0.1 (*q* value).

### In silico validation and analysis

A search of the Gene Expression Omnibus (GEO, www.ncbi.nlm.nih.gov/geo) database was conducted to identify publicly available datasets of microarray gene expression experiments previously performed with human preimplantation embryos. This search identified a similarly designed study, with array data submitted to GEO under the accession number GSE18290 [4]. In that study, total RNA from preimplantation embryos (from single cell to blastocyst stage) was extracted, amplified, and hybridized onto Affymetrix U133 Plus 2.0 Arrays (Affymetrix). From the 52 datasets submitted by these investigators (16 bovine, 18 mouse, and 18 human), we used those six representing the embryos of interest for our study (all three human embryos at the eight-cell stage and all three human embryos at the blastocyst stage).

Gene expression data were analyzed using comparisons identical to our original discovery set. Only genes identified as significant in the discovery set were used for validations; a two-sample *t* test, with significance at *p* <0.05, was used for comparisons. For comparisons, we used the Biometric Research Branch (BRB) ArrayTools software suite (version 2.13.2 for X64 systems), an integrated package for visualization and statistical analysis utilizing Microsoft Excel at the front end, with tools developed in the R statistical system.

### In vitro validation and analysis

Eight genes of interest, based on potential mechanism and fold change, were selected for validation in vitro using reverse transcription quantitative PCR (RT-qPCR). RNA was purified from individual embryos (six D3 embryos and six D5 embryos for each RT-qPCR assay) using the PicoPure RNA Isolation kit (Arcturus) according to the protocol with the addition of a DNase I digest step (#79254, Qiagen) prior to wash buffer 2 and a final elution volume of 20 μL. The eluent was concentrated into a volume of 10 μL by SpeedVac (Thermo Fisher). This method of embryonic RNA isolation was confirmed by RT-qPCR assay for the embryo-specific genes *OCT4* and *NANOG* (data not shown). Reverse transcription was performed using a High Capacity cDNA Reverse Transcription Kit (#4368814, Life Technologies). Gene-specific RT-qPCR assays were carried out in triplicate using prevalidated TaqMan Assays (Life Technologies). Each embryo had sufficient RNA to be used for two gene-specific assays plus control. No total RNA amplifications were carried out in order to avoid the possibility of introducing amplification bias into the study.

### Immunofluorescence

Previously cryopreserved blastocyst-stage embryos were thawed, allowed to re-expand for 4 h in culture media, and washed in a solution of Gibco Dulbecco’s phosphate-buffered saline (PBS, Life Technologies) with added 1 mg/mL polyvinylpyrrolidone (PVP, Sigma). Embryos were fixed with 4 % paraformaldehyde for 30 min at room temperature, washed three times in PVP/PBS, and permeabilized in 0.2 % Triton X-100 in PVP/PBS for 30 min at room temperature. Blocking was performed overnight at 4 °C using 10 % normal goat sera (G-9023, Sigma) in PVP/PBS. The following primary antibodies were prepared in 10 % normal goat sera: rabbit anti-S100A14 IgG at 1/200 dilution (10489-1-AP, Proteintech) and rabbit anti-S100A16 IgG at 1/100 dilution (11456-1-AP, Proteintech). Embryos were incubated in one of these two antibodies, no-antibody control (10 % normal goat sera), or normal rabbit IgG (011-000-003, Jackson Immuno Research) prepared to equal concentration as the antibodies (for anti-S100A14 control, 1.6 μg/mL; for anti-S100A16 control, 2.5 μg/mL). Embryos were incubated overnight at 4 °C, washed three times in PVP/PBS, and incubated in F(ab’)2-goat anti-rabbit IgG (H + L) secondary antibody, Alexa Fluor® 488 conjugate (A11070, Life Technologies), diluted 1/500 in 10 % normal goat sera, for 4 h at room temperature. Embryos were washed in PVP/PBS, put through a dilution series of 25 %, 50 %, 75 %, and 100 % Vectashield with 4′,6-diamidino-2-phenylindole (DAPI) (Life Technologies) in PVP/PBS as described elsewhere [15], and mounted in a hanging-drop slide to preserve blastocyst structure. Embryos were imaged using a Zeiss LSM 710 confocal laser scanning microscope (Zeiss, Germany).

### Statistical analysis

For Affymetrix arrays in the discovery set, two-sample *f* tests were used to determine differential gene expression with a univariate significance level of *p* <0.001. For in silico validation, a two-sample *t* test was used to determine the significance of gene expression, with a significance set at *p* <0.05. The agreement between differentially expressed genes in both sets (discovery and validation in silico) was evaluated by Cohen’s kappa. Test κ values are interpreted as <0 (no agreement), 0–0.20 (slight), 0.21–0.40 (fair), 0.41–0.60 (moderate), 0.61–0.80 (substantial or good), and 0.81–1 (almost perfect agreement). For in vitro validation, all raw expression data (Ct values) were normalized (ΔCt) using the endogenous RNA control 18S rRNA. Gene expression differences between D3 and D5 embryos were assessed as fold change via the conventional ΔΔCt method [16, 17]. Statistical significance was evaluated with a two-sample *t* test with unequal variances, with *p* values <0.05 considered significant.

## Results

### Discovery of differentially expressed genes

Affymetrix microarray analysis of three good-quality D3 embryos and three good-quality D5 embryos identified differential expression of 51 genes. Of these, 31 were overexpressed in D5 blastocyst-stage embryos and 20 were underexpressed (Supplemental Table 1). Pathway analyses of these genes using KEGG found five overrepresented pathways including amoebiasis and cancer (Table 1).

**Table 1:**
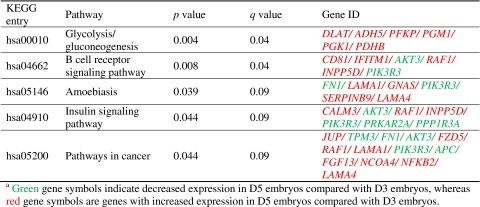
Pathways enriched by genes differentially expressed in the discovery set

### In silico validation

We identified a similar study of human preimplantation embryos through the NCBI Gene Expression Omnibus and compared our 51 differentially expressed genes with 47 that were available for comparison. Of these, 23 genes were statistically significant in both sets, and 21 of 23 displayed expression congruent with our original findings (Table 2). This represents a κ value equal to 0.8 (substantial or good correlation).

**Table 2:**
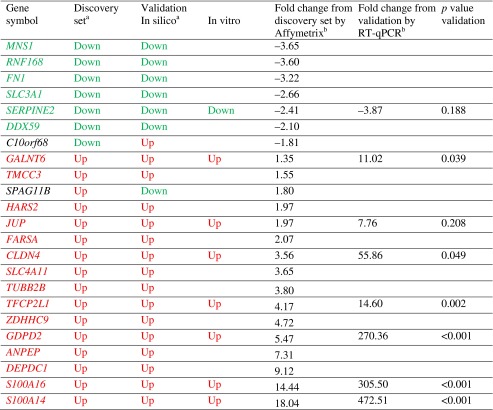
In silico and in vitro validation of differentially expressed genes in the human blastocyst-stage embryos

^a^κ = 0.8 between the discovery set and in silico validation

^b^ *r* = 0.92 between the fold change from discovery set and the fold change from in vitro validation (*p* = 0.006; *df* = 6)

### In vitro validation

Based on potential biological significance in embryo adhesion and invasion as well degree of fold change, we chose eight genes to validate in vitro, using an independent panel of six D3 and six D5 embryos for each gene assay. Among the eight chosen genes, all displayed differential expression patterns consistent with the original array and in silico validation results and six achieved statistical significance (Table 2). The overall correlation between fold changes from our Affymetrix arrays and TaqMan gene-specific assays was *r* = 0.92 (*p* = 0.006; *df* = 6).

### Immunofluorescence and localization of S100 proteins

**Fig. 1:**
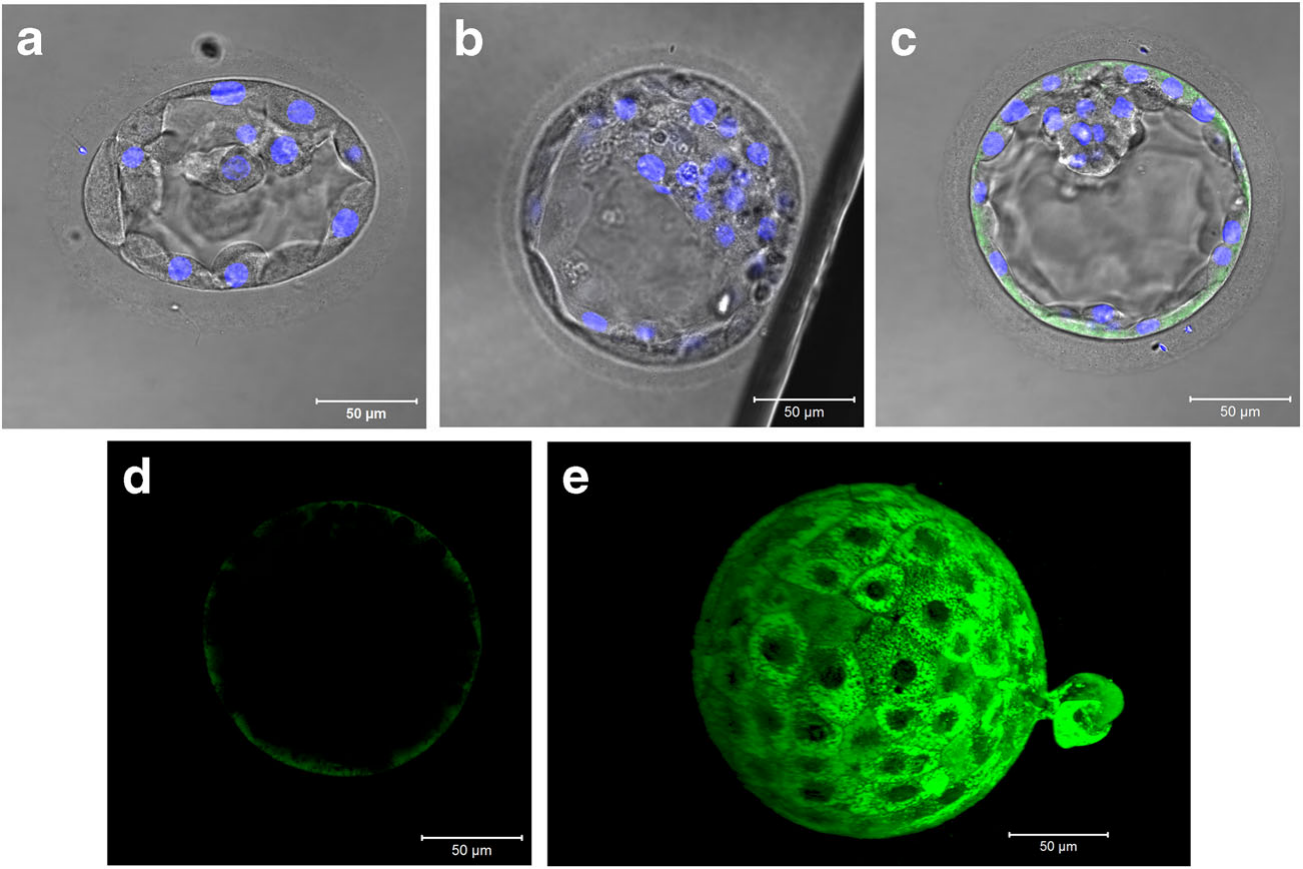
Immunolocalization of S100A14 to the trophectoderm of human blastocyst-stage embryos. Representative images taken for immunofluorescence with DAPI for nuclear stain. **a** No primary antibody, **b** rabbit IgG control, **c** 1:100 anti-S100A14 (bright-field overlay), **d** Alexa Fluor® 488 only, and **e** 3D reconstruction (Z stack) of S100A14 with embryo hatching at approximately 4 o’clock

**Fig. 2:**
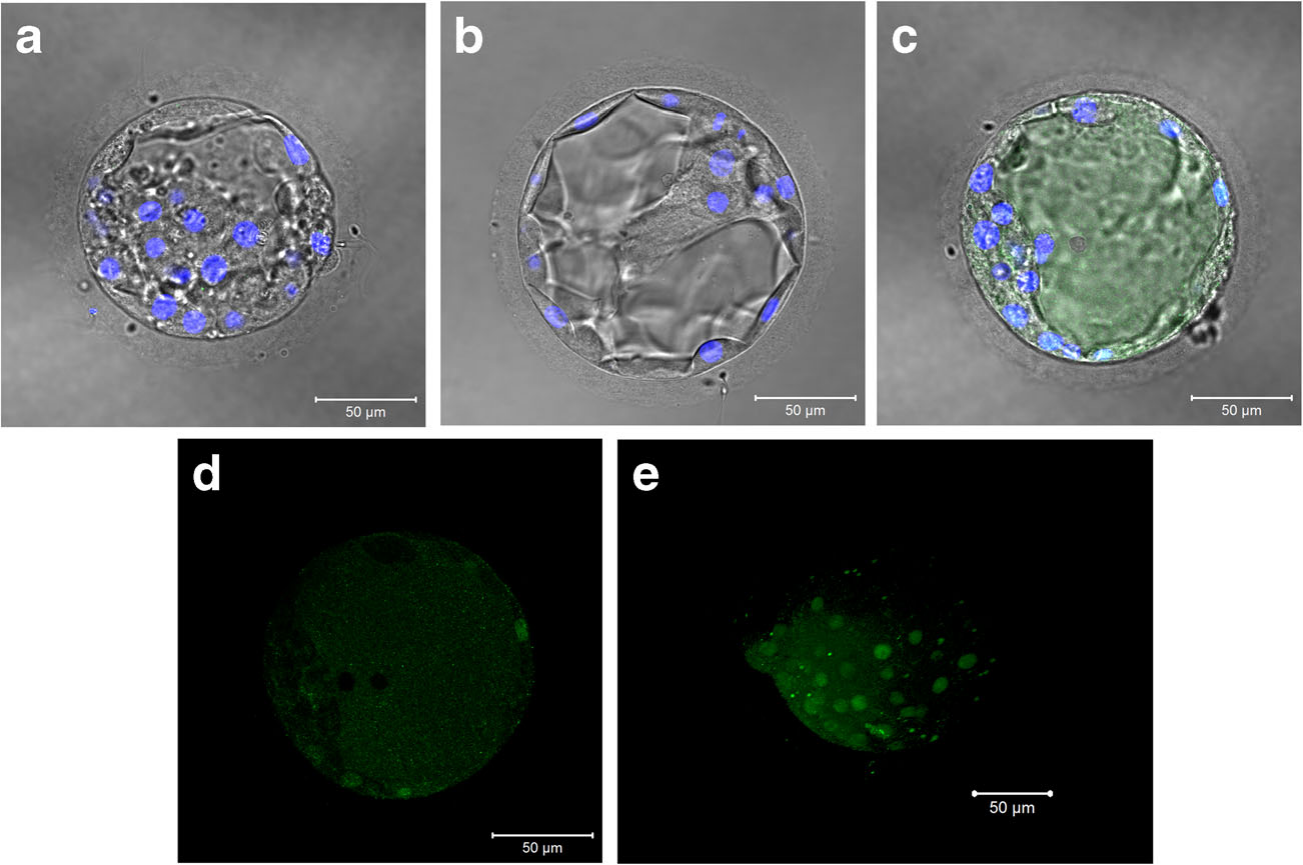
Immunolocalization of S100A16 suggesting embryo polarity and expression near the hatching portion of human blastocyst-stage embryos. Representative images taken for immunofluorescence with DAPI for nuclear stain. **a** No primary antibody, **b** rabbit IgG control, **c** 1:100 anti-S100A16 (bright-field overlay), **d** Alexa Fluor® 488 only, and **e** 3D reconstruction (Z stack) of S100A16 with embryo hatching at approximately 9 o’clock

The two genes with the greatest differential expression levels, S100A16 and S100A14, were selected for localization studies. Using confocal microscopy, we observed that S100A14 is exclusively localized to the trophectoderm cells and is absent in the inner cell mass (Fig. 1). Further, the intensity of the staining appears greater in trophectoderm cells nearer to the pole closest to the trophectoderm hatching point (Fig. 1e) but appears to be present in all cells. S100A16 was also seen in the trophectoderm (Fig. 2), was more finely distributed in expression, and was lower in intensity in the inner cell mass (Fig. 2d). Expression of this protein appeared to be limited to some nuclei and also polarized to one quadrant of the trophectoderm close to the trophectoderm hatching point (Fig. 2e).

## Discussion

Our study shows that there are numerous differentially expressed genes in human blastocyst-stage embryos compared with cleavage-stage embryos. These genes are involved in cellular movement and cancer pathways and are likely necessary for embryo adhesion and implantation. We report here for the first time that S100A14 and S100A16, which have been previously shown to be associated with cancer cell invasion [18–22], are abundant in blastocysts and appear to be localized to the trophectoderm, consistent with a role in the invasiveness of the trophoblast during implantation.

In 2009, Zhang and colleagues reported that human embryo preimplantation development could be divided into two transitions: early versus late maternal genomic expression (from oocyte to four-cell embryo) and embryonic genomic expression (eight-cell embryo to blastocyst stage), with most differentially expressed embryonic genes involved in metabolic processes [7]. Similarly, we identified increased expression of embryonic genes overrepresented in pathways related to glycolysis and dynamic expression of genes in insulin signaling, and both pathways are important to the developing embryo. Additionally, we are the first to report differential expression of embryonic genes involved in pathways related to amoebiasis and cancer, processes likely important for rapid cell division, movement, adhesion, and invasion, all which must occur during early embryo development and implantation.

Twenty-one of our original discovery set of 51 differentially expressed genes were validated in silico by comparison to Affymetrix data published by Xie and colleagues, with global transcriptional similarities noted among bovine, murine, and human embryos, suggesting their evolutionary importance [4]. We chose to validate eight of our genes further via RT-qPCR and found that all eight exhibited changes in a manner consistent with the in silico validations and, of these, six were statistically significant in our validation assays.

Polypeptide *N*-acetylgalactosaminyltransferase 6 (known as GALNT6) is a member of the GALNT family of proteins which begins mucin-type O-linked protein glycosylation [23]. GALNT6 may be involved with the synthesis of oncofetal fibronectin, which is secreted by cultured trophoblasts [24] and may potentially facilitate embryo adhesion to the uterine endometrium [25]. Additionally, alterations in mucin O-glycosylation are implicated in malignant transformation of some carcinomas; GALNT6 expression has been found to be greater in breast cancer tissue compared with normal breast tissue [26] and was correlated with venous invasion in gastric cancers [27].

Claudin 4 is a member of the claudin protein family that plays a ubiquitous and integral role in the formation of gap junctions. Claudin 4 is necessary for appropriate structure and function of reproductive tissues, such as the blood-testis barrier [28], and its messenger RNA (mRNA) levels in the luteal-phase endometrium have been correlated with pregnancy [29]. Mouse embryos cultured in the presence of *Clostridium perfringens* enterotoxin inhibitory to claudin 4 failed to form a mature blastocele cavity, demonstrating the importance of claudin 4 in the normal formation of blastocysts [30]. In addition to tight junctions, claudin 4 has been shown to be localized in cellular projections of breast cancer cells, where it promotes cellular motility in a wound-healing model [31]. Thus, its increased level in our blastocyst-stage embryos is suggestive of not only its importance in blastocyst formation but could be potentially involved in uterine implantation.

Perhaps the most important finding reported here is the 300–400-fold increase in the mRNA expression of two S100 calcium-binding proteins A16 and A14, with immunofluorescence documenting the predominant expression of these two proteins within the trophectoderm of the human blastocyst. S100A14, a 104-amino acid protein with calcium-binding motifs, was shown to be overexpressed in 10 tumor types (including ovary, breast, and uterus), but not present in normal placenta [32]. Its expression has been detected in the circulating stem cells of patients with advanced metastatic colorectal and breast cancer [18]. S100A14 was also associated with the capability of breast cancer cells to produce distant metastases [19] and with increased motility and invasiveness of malignant cells in colon cancer cell lines [20]. Further, it has been shown to co-localize with HER2, a known EGFR protein associated with more aggressive breast cancers [33]. We found that S100A14 localizes to the trophectoderm of blastocyst-stage embryos with a slightly greater expression in the pole of the blastocyst hatching through the zona pellucida (Fig. 1e). S100A14 overexpression is known to modulate expression of matrix metalloproteinase 2 (MMP2), a key factor in the implantation process [20]. Our RT-qPCR validation and immunofluorescence localization to the trophectoderm, coupled with S100A14’s function in aggressive cancer mechanisms and regulation of expression of a key gene, MMP2, involved in implantation suggests that S100A14’s role in the implantation process needs to be further examined.

S100A16, also a calcium-binding protein, is mainly localized in nucleoli, with nucleocystoplasmic transport stimulated by high levels of intracellular calcium levels [34]. It is co-expressed with S100A14 in oral squamous cell cancer, breast cancer, and several cancer cell lines [21, 22]. Interestingly, we noted differences in signal intensity in our immunofluorescence studies on S100A16 suggestive of embryo polarity as we captured more diffuse signal in the quadrant of the trophectoderm near the hatching point. For the remainder of the trophectoderm, only the nucleoli appear to be positive for S100A16 (Fig. 2e). Previous studies have shown that cellular stretch can cause intracellular calcium influx via stretch-activated calcium channels [35]. If calcium influx is occurring at the embryo hatching point, then this would explain the more diffuse localization of S100A16 outside the nucleoli and into the cytoplasm, as seen with calcium influx within mouse glial cells [34].

We also examined potential roles for transcription factor CP2-like 1 (TFCP2L1) and glycerophophodiester phosphodiesterase domain containing 2 (GDPD2); however, since these genes have not yet been well described in the literature, we were unable to draw meaningful conclusions about their potential involvement in embryonic development.

The work presented here raises questions to be answered in subsequent studies. Several papers have suggested that bovine embryos, having similar gene expression profiles to humans, are good models for human embryonic development and have shown differential gene expression at different stages of preimplantation development [36–38]. In examining these reports, we found that 15 of our 51 differentially expressed genes were differentially expressed in at least one of those studies, and five of those were among the six genes we validated by RT-qPCR (*CLDN4*, *TFCP2L1*, *GDPD2*, *S100A16*, and *S100A14*). Additionally, the two S100 proteins were identified by Jiang et al. [37] as being represented in Gene Ontology pathways of cellular motility, migration, and chemotaxis, which is consistent with our discovery set pathway analyses. As is common in most other fields of study, critically important genes may overlap in multiple putative pathways (Table 1), which may affect their degree of overrepresentation. Recognizing the importance of embryonic research and scarcity of human embryos available, we suggest a future study of these genes, and others, which could be performed in bovine preimplantation embryos, to include protein expression and localization studies as well to independently validate these pathways described.

Our studies were carried out exclusively on previously cryopreserved embryos, and there may be differences in gene expression in fresh versus cryopreserved embryos. However, cryopreserved embryos have been shown to be a more consistent, reliable source for gene expression studies as compared with fresh embryos [39]. Differences in gene expression can also be associated with different culture systems, as has been previously shown in other animal species [40, 41] and, recently, in human preimplantation embryos [42]. Given that we were able to validate our initial findings in a dataset obtained from embryos cultured in a different system, we suggest that the gene expression patterns we have observed may be important enough to be conserved in the embryo’s developmental processes independent of culture environment. Finally, while genes expressed in preimplantation embryonic development may be important in processes leading to pregnancies and live births, our results cannot yet be matched with these clinically important outcomes. We hope, however, that with this knowledge, we will be able to characterize genetic signatures, via trophectoderm biopsies obtained during preimplantation genetic screening, that are associated with intrauterine pregnancies and live births. Eventually, such information may have an important translational value by incorporating this information into both invasive and non-invasive criteria for embryo selection, beyond just chromosome number, thus allowing for selection of euploid embryos with the best chance of implantation.

In summary, we present a panel of genes differentially expressed in blastocyst-stage embryos relative to cleavage-stage embryos. Further, we define their association with pathways related to cellular movement and cancer mechanisms and localize two of the expressed proteins to the trophectoderm. We now open a window of opportunity for clinicians and basic scientists to better understand the molecular mechanisms in determining embryonic competence and potentially develop means to improve in vitro fertilization practices by selecting the most capable embryos for transfer.

## Electronic supplementary material

Below is the link to the electronic supplementary material.

ESM 1 (DOCX 21 kb)
