# Supplementary material for: Differentially expressed genes in preimplantation human embryos: potential candidate genes for blastocyst formation and implantation
Source: J Assist Reprod Genet. 2016 May 30;33(8):1017–25. doi: 10.1007/s10815-016-0745-x (PMC4974233; doi:10.1007/s10815-016-0745-x)
Supplement: Supplementary file 1 — (DOCX 21 kb) [file 10815_2016_745_MOESM1_ESM.docx]

**Supplemental Table 1:** Differentially expressed genes in blastocyst embryos – discovery set

| **Gene Symbol** | **Gene Name** | **Chromosome Location** | **Fold Change**  **(D5 vs. D3)** | **p value ^a^** |
| --- | --- | --- | --- | --- |
| *CEP170* | Centrosomal Protein 170kDa | 1q44 | -5.75 | 5.29 x 10^-5^ |
| *SUPT3H* | Suppressor Of Ty 3 Homolog | 6p21.1-p21.3 | -4.33 | 2.51 x 10^-4^ |
| *MNS1* | Meiosis-Specific Nuclear Structural 1 | 15q21.3 | -3.65 | 4.82 x 10^-4^ |
| *RNF168* | Ring Finger Protein 168, E3 Ubiquitin Protein Ligase | 3q29 | -3.60 | 1.98 x 10^-4^ |
| *HBP1* | HMG-Box Transcription Factor 1 | 7q22.3 | -3.42 | 2.50 x 10^-4^ |
| *FN1* | Fibronectin 1 | 2q34 | -3.22 | 6.63 x 10^-4^ |
| *SLU7* | SLU7 Splicing Factor Homolog | 5q33.3 | -2.94 | 2.48 x 10^-4^ |
| *ZFP82* | ZFP82 Zinc Finger Protein | 19q13.12 | -2.87 | 5.26 x 10^-4^ |
| *SLC3A1* | Solute Carrier Family 3 (Amino Acid Transporter Heavy Chain), Member 1 | 2p16.3 | -2.66 | 6.94 x 10^-4^ |
| *FAM53C* | Family With Sequence Similarity 53, Member C | 5q31 | -2.54 | 7.14 x 10^-4^ |
| *RFC1* | Replication Factor C (Activator 1) 1, 145kDa | 4p14-p13 | -2.43 | 2.45 x 10^-4^ |
| *SERPINE2* | Serpin Peptidase Inhibitor, Clade E, Member 2 | 2q36.1 | -2.41 | 5.35 x 10^-4^ |
| *KDM4D* | Lysine (K)-Specific Demethylase 4D | 11q21 | -2.40 | 6.70 x 10^-4^ |
| *RTN3* | Reticulon 3 | 11q13 | -2.18 | 6.24 x 10^-4^ |
| *PUM2* | Pumilio RNA-Binding Family Member 2 | 2p24.1 | -2.11 | 1.87 x 10^-4^ |
| *DDX59* | DEAD (Asp-Glu-Ala-Asp) Box Polypeptide 59 | 1q32.1 | -2.10 | 1.93 x 10^-4^ |
| *C10orf68* | Chromosome 10 Open Reading Frame 68 | 10p11.22 | -1.81 | 8.63 x 10^-4^ |
| *RBM33* | RNA Binding Motif Protein 33 | 7q36.3 | -1.77 | 9.27 x 10^-4^ |
| *TPM3* | Tropomyosin 3 | 1q21.2 | -1.53 | 3.06 x 10^-4^ |
| *SERTAD4* | SERTA Domain Containing 4 | 1q32.1-q41 | 1.31 | 1.97 x 10^-4^ |
| *GALNT6* | Polypeptide N-Acetylgalactosaminyltransferase 6 | 12q13 | 1.35 | 5.09 x 10^-4^ |
| *TMCC3* | Transmembrane And Coiled-Coil Domain Family 3 | 12q22 | 1.55 | 5.08 x 10^-4^ |
| *AGTR1* | Angiotensin II Receptor, Type 1 | 3q24 | 1.58 | 6.54 x 10^-4^ |
| *C18orf2* | Charged Multivesicular Body Protein 1B | 18p11.21 | 1.58 | 8.07 x 10^-4^ |
| *RPN1* | Ribophorin I | 3q21.3 | 1.77 | 2.78 x 10^-4^ |
| *ESRRB* | Estrogen-Related Receptor Beta | 14q24.3 | 1.78 | 8.28 x 10^-4^ |
| *PNMA6A* | Paraneoplastic Ma Antigen Family Member 6A | Xq28 | 1.80 | 1.19 x 10^-4^ |
| *SPAG11B* | Sperm Associated Antigen 11B | 8p23.1 | 1.80 | 3.26 x 10^-4^ |
| *MGC5590* | Small Integral Membrane Protein 2 | 13q14.11 | 1.82 | 4.78 x 10^-4^ |
| *STK16* | Serine/Threonine Kinase 16 | 2q35 | 1.84 | 7.17 x 10^-4^ |
| *HARS2* | Histidyl-TRNA Synthetase 2, Mitochondrial | 5q31.3 | 1.97 | 5.97 x 10^-4^ |
| *JUP* | Junction Plakoglobin | 17q21 | 1.97 | 1.96 x 10^-4^ |
| *FARSA* | Phenylalanyl-TRNA Synthetase, Alpha Subunit | 19p13.2 | 2.07 | 8.56 x 10^-4^ |
| *SFXN4* | Sideroflexin 4 | 10q26.11 | 2.37 | 8.76 x 10^-4^ |
| *SLC36A4* | Solute Carrier Family 36 (Proton/Amino Acid Symporter), Member 4 | 11q21 | 2.39 | 2.12 x 10^-4^ |
| *NEK9* | NIMA-Related Kinase 9 | 14q24.3 | 2.44 | 8.90 x 10^-4^ |
| *ERBB3* | Erb-B2 Receptor Tyrosine Kinase 3 | 12q13 | 2.62 | 3.39 x 10^-4^ |
| *ABHD10* | Abhydrolase Domain Containing 10 | 3q13.2 | 3.09 | 9.98 x 10^-4^ |
| *TMEM133* | Transmembrane Protein 133 | 11q22.1 | 3.26 | 3.66 x 10^-4^ |
| *CLDN4* | Claudin 4 | 7q11.23 | 3.56 | 7.37 x 10^-4^ |
| *SLC4A11* | Solute Carrier Family 4, Sodium Borate Transporter, Member 11 | 20p12 | 3.65 | 4.75 x 10^-4^ |
| *SLC25A11* | Solute Carrier Family 25 (Mitochondrial Carrier; Oxoglutarate Carrier), Member 11 | 17p13.3 | 3.69 | 6.45 x 10^-4^ |
| *TUBB2B* | Tubulin, Beta 2B Class IIb | 6p25 | 3.80 | 8.07 x 10^-4^ |
| *TFCP2L1* | Transcription Factor CP2-Like 1 | 2q14 | 4.17 | 2.36 x 10^-4^ |
| *ZDHHC9* | Zinc Finger, DHHC-Type Containing 9 | Xq26.1 | 4.72 | 4.37 x 10^-4^ |
| *GDPD2* | Glycerophosphodiester Phosphodiesterase Domain Containing 2 | Xq13.1 | 5.47 | 3.42 x 10^-4^ |
| *ANPEP* | Alanyl (Membrane) Aminopeptidase | 15q25-q26 | 7.31 | 5.36 x 10^-4^ |
| *DEPDC1* | DEP Domain Containing 1 | 1p31.2 | 9.12 | 5.84 x 10^-4^ |
| *S100A16* | S100 Calcium Binding Protein A16 | 1q21 | 14.44 | 5.63 x 10^-4^ |
| *S100A14* | S100 Calcium Binding Protein A14 | 1q21.3 | 18.04 | 4.49 x 10^-4^ |

^a^ All candidate genes were selected with a strict criteria of significance (p < 0.001) due to the multiple comparisons effect.

**Supplemental video:**

Video 1: S100A14 images in 3D rotation

Video 2: S100A16 images in 3D rotation
